# Same same but different: Subtle but consequential differences between two measures to linearly integrate speed and accuracy (LISAS vs. BIS)

**DOI:** 10.3758/s13428-022-01843-2

**Published:** 2022-05-20

**Authors:** Heinrich R. Liesefeld, Markus Janczyk

**Affiliations:** Department of Psychology, University of Bremen, Hochschulring 18, D-28359 Bremen, Germany

**Keywords:** Speed–accuracy trade-off, Methods in experimental psychology, Integration of errors and response times, Repeated-measures designs

## Abstract

Condition-specific speed–accuracy trade-offs (SATs) are a pervasive issue in experimental psychology, because they sometimes render impossible an unambiguous interpretation of experimental effects on either mean response times (mean RT) or percentage of correct responses (PC). For between-participants designs, we have recently validated a measure (*Balanced Integration Score*, *BIS*) that integrates standardized mean RT and standardized PC and thereby controls for cross-group variation in SAT. Another related measure (*Linear Integrated Speed–Accuracy Score, LISAS*) did not fulfill this specific purpose in our previous simulation study. Given the widespread and seemingly interchangeable use of the two measures, we here illustrate the crucial differences between LISAS and BIS related to their respective choice of standardization variance. We also disconfirm the recently articulated hypothesis that the differences in the behavior of the two combined performance measures observed in our previous simulation study were due to our choice of a between-participants design and we demonstrate why a previous attempt to validate BIS (and LISAS) for within-participants designs has failed, pointing out several consequential issues in the respective simulations and analyses. In sum, the present study clarifies the differences between LISAS and BIS, demonstrates that the choice of the variance used for standardization is crucial, provides further guidance on the calculation and use of BIS, and refutes the claim that BIS is not useful for attenuating condition-specific SATs in within-participants designs.

Since the early studies by Woodworth (1899) it is well established that performing something faster comes at the cost of less accuracy (see also Fitts, 1954, and many others). This observation has become known as the *speed–accuracy trade-off* (*SAT*; for reviews, see Heitz, 2014; Wickelgren, 1977). Interesting in itself as a topic of research (e.g., Fiedler et al., 2020; Hedge et al., 2019), an SAT can also cause interpretational problems in studies assessing mean response times (mean RT) or the percentage of correct responses (PC) as the main dependent variable(s).

More precisely, participants in such studies are typically confronted with a conundrum: they are asked to perform the task “as fast *and* as accurately as possible,” “as fast as possible without sacrificing accuracy,” and the like. What is more important according to such instructions, speed or accuracy? And how low can PC fall and still count as not “sacrificing accuracy”? As instructions do not provide answers to these questions, participants must answer them for themselves. In other words, because responding faster necessarily incurs a higher risk of committing an error, participants always have to decide for some trade-off between speed and accuracy. The relation between speed and accuracy on this continuum has, for example, been described as an exponential approach to a limit that follows the form

$$\mathrm{PC}=\left\{\begin{array}{l}\quad\quad\quad\quad\quad\quad50\;\mathrm{if}\;\overline{\mathrm{RT}}<\mathrm\delta\;\\\frac\lambda2\cdot\begin{bmatrix}1-e^{-\gamma\cdot\left(\overline{RT}-\delta\right)}\end{bmatrix}\;+\;50\;\mathrm{if}\;\overline{\mathrm{RT}}\;\geq\mathrm\delta\end{array}\right.$$

where $\overline{RT}$ is mean RT, δ is the *x*-offset, γ the steepness of the curve, and λ the PC asymptote (see Wickelgren, 1977; see also Usher & McClelland, 2001, and for a broader discussion, see Luce, 1986). An example is visualized in Fig. 1. Up to a certain mean RT level (200 ms in the example), mere guessing takes place and PC remains at about 50% (assuming two response alternatives with one being the correct one, thus a two-alternative forced-choice task). With increasing mean RT, then, PC increases as well until an asymptotic level is reached. What becomes clear from this visualization is that SAT is conceived of as a unidimensional phenomenon: Each point on the curve refers to one particular setting on the SAT and a change in SAT affects mean RT and PC at the same time (see Appendix 1 for an alternative view).

**Fig. 1:**
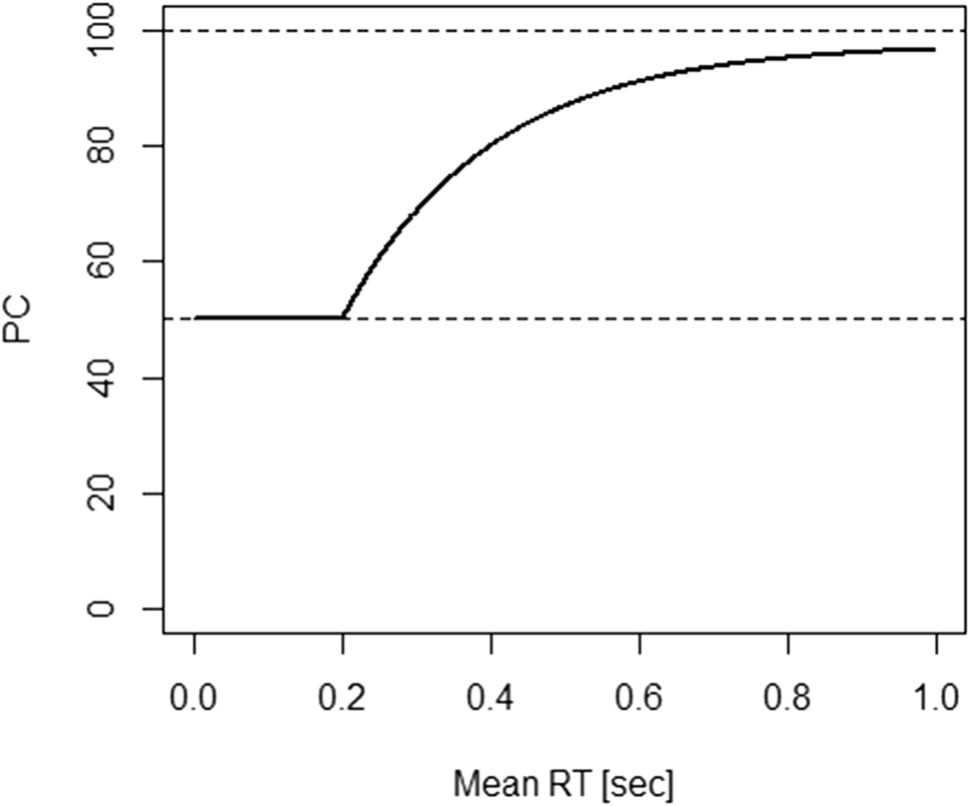
Illustration of a speed–accuracy curve with δ = 0.20, γ = 5, and λ = 95 (adopted from Wickelgren, 1977)

The issue of uncontrolled SATs in psychological studies is most evident when comparing groups of participants: due to differences in their personality (e.g., when comparing age groups) or due to differences between conditions (e.g., different stimuli or instructions), one group might—on average—choose a different SAT than the other group and therefore perform faster and less accurately or vice versa, even if average ability and/or task difficulty is comparable across groups. The study by Liesefeld and Janczyk (2019) suggests that out of several available measures to combine mean RT and PC, the Balanced Integration Score (BIS; Liesefeld et al., 2015) works best for solving this issue in between-participants designs. This measure attenuates variations in SAT better than other measures that have been used for this purpose (*Inverse Efficiency Score* and *Rate Correct Score*; Akhtar & Enns, 1989; Bruyer & Brysbaert, 2011; Townsend & Ashby, 1983; Woltz & Was, 2006), including a more recently developed measure, the goal of which is similar to that of BIS: integrating speed and accuracy in a balanced manner. This alternative measure has been termed the *Linear Integrated Speed–Accuracy Score* (*LISAS*; Vandierendonck, 2017, 2018, 2021b).

BIS combines mean RT and PC according to the following formula (Liesefeld & Janczyk, 2019):1where ${z}_{i,j}^x$ is the *z*-standardized^1^ performance (mean RT or PC, respectively) for participant *i* in condition *j*, ${S}^{\overline{RT}}$ refers to the standard deviation (SD) of mean RTs used in the calculation of BIS,  refers to the grand mean RT—that is, the average of mean RTs of all conditions and participants—and $\overline{PC}$ refers to the average of PCs of all conditions by participant combinations. Importantly, *z* standardization is based on the variance across *averaged* data points (mean RT and PC), that is, on those data points that would also go into a standard *t* test or analysis of variance (ANOVA), rather than the variance across individual trials (RT and accuracy). Typically, this standardization is performed across all cells of the design (e.g., *n* × *J* data points in a one-way ANOVA with *J* conditions and *n* participants per condition). As demonstrated below, it turns out to be crucial that the variance for the standardization comes from the aggregated data, that is, to use the standard deviations across mean RTs and PCs rather than the standard deviations across trials.

LISAS is calculated according to the following formula^2^:

2 $${LISAS}_{i,j}={\overline{RT}}_{i,j}+\frac{S_i^{\overline{RT}}}{S_i^E}\cdot{PE}_{i,j}$$

where *S*^RT^ refers to the SD of RTs across trials and *S*^E^ refers to the SD of errors across trials (which equals $\sqrt{PE\left(1- PE\right)}$). Thus, in contrast to BIS, LISAS uses the SDs across trials for individual participants, but otherwise the intention of the two measures is similar: mean RT and percentage of errors (PE) (= 1 − PC) is brought to the same scale and added up (see Vandierendonck, 2021b, Appendix A). Yet, as will be demonstrated below, the choice of the SD is crucial for how the respective measure behaves with regard to SATs. Vandierendonck has used two versions of the formula, one where S^RT^ and S^E^ are calculated across all conditions of a given participant (which we assume is the default and which is displayed in Eq. 2; Vandierendonck, 2017, 2021b) and one where S^RT^ and S^E^ are calculated separately per condition and participant (Vandierendonck, 2018; which in the following we refer to as LISAS^cond^ as a shorthand for condition-specific LISAS).^3^

Given the widespread use of within-participants designs in behavioral research and the frequent use of LISAS and BIS in within-participants comparisons, including many studies in which we have been involved (e.g., Allenmark et al., 2019; Barrientos et al., 2020; Bratzke & Ulrich, 2021; Chen et al., 2021; English et al., 2021; Liesefeld et al., 2015, 2019; Liesefeld & Müller, 2021; Madrid & Hout, 2019; Mueller et al., 2020; Schuch & Pütz, 2021; Serrien & Spapé, 2021; Smith et al., 2019), it is important to note that LISAS was explicitly developed for the within-participants case (Vandierendonck, 2021b, p. 22). By contrast, BIS is by no means restricted to within-participants designs, but we and others consider many use cases even going beyond experimental psychology (e.g., Bakun Emesh et al., 2021; Draheim et al., 2019; Liesefeld & Janczyk, 2019; Liu et al., 2019; Mueller et al., 2019; Palmqvist et al., 2020; Stojan et al., 2021; Unsworth et al., 2020; White et al., 2021). This difference in scope of the two measures, in retrospect, also implies that our previous comparison of BIS and LISAS based on a between-participants design might not have been the fairest case (see Vandierendonck, 2021b, p. 22). To make up for this, Vandierendonck (2021b) has recently validated and compared the two measures on data explicitly simulated to conform to a typical within-participants design, concluding that the two measures behave highly similar and neither of them satisfactorily attenuates variations in SATs in this case. By contrast, the present study provides first evidence that BIS (but not LISAS) fulfills this purpose very well. These opposing conclusions can be traced back to various consequential mistakes in Vandierendonck’s analyses, which we correct for in reanalyses of one of his simulated data sets. We also point out problems with the simulations reported in Vandierendonck (2021b) and clarify several additional points that have been brought up since the publication of Liesefeld and Janczyk (2019). Although it does not aim to provide a comprehensive validation of combined measures in within-participants designs, the present paper demonstrates the differences between LISAS and BIS from various perspectives, thereby informing the choice between these two seemingly similar measures. Along the way, we also offer advice on how to avoid various pitfalls in the calculation of BIS and in the simulation of within-participants data.

## Simulating differential speed–accuracy trade-offs in within-participants designs

To explore how a given measure handles variation in SATs, it is useful to produce data for which variations in SATs are known a priori. As there currently is no undisputed experimental method of inducing specific levels of SAT and as developing, validating, and using such a method is highly resource intensive, simulating data with an established mathematical model of human performance seems the most straightforward and efficient first step to tackle this question.

From among the many cognitive models that would fulfill this purpose, Liesefeld and Janczyk (2019) used a relatively simple version of the drift-diffusion model (Ratcliff, 1978; Ratcliff et al., 2016; for a similar approach, see Dutilh et al., 2012; Hedge et al., 2018a, b, 2021; Lerche & Voss, 2018; Vandierendonck, 2021b). This model simulates a decision process, assuming that, from a starting point *z*, evidence for the correct response continuously and noisily accumulates with a certain drift rate *v* until a preset threshold *a* is reached, thus producing a correct response. Because of the noise, typically modelled as a scaled Wiener process, the activation reaches the lower threshold at zero by chance on some trials, thus producing an incorrect response.^4^ Increasing the value of *v de*creases mean RT and *in*creases PC at the same time and is thus often thought to reflect decreases in task difficulty or increases in cognitive ability. By contrast, increasing the value of *a* (i.e., increasing the distance between the upper and lower threshold and thereby increasing the distance of the starting point to the thresholds as well) *in*creases mean RT and PC at the same time (see also Lerche & Voss, 2018), thus capturing changes on the SAT continuum towards a more conservative responding. As such, this model is suited to simulate variations in SAT and difficulty/ability independently by variations in *a* and *v*, respectively.

Arbitrary as this selection might be, the drift-diffusion model has several characteristics that are highly desirable for our purposes: (a) It makes predictions on mean and trial-wise RTs and accuracies, (b) the model is widely used and is well established in terms of being able to account for empirical data from a huge range of cognitive tasks, and (c) there are separate parameters that can be interpreted as reflecting SAT settings (threshold separation *a*) or difficulty (drift rate *v*).

To see how simulations need to be adapted for the present purposes (in comparison to Liesefeld & Janczyk, 2019), it is necessary to consider what differentiates a between-participants from a within-participants design and how that affects the data. The core feature of within-participants designs is that the same participant performs both (or all) conditions and that each participant is compared to themselves via, for example, repeated-measures ANOVAs or paired *t* tests. This ensures that pre-experimental interindividual variability (between-participants variance) does not affect the error term of significance tests (the participant × condition interaction) and thereby typically increases their statistical power. As this pre-experimental variability is the same in all conditions, performance across conditions is highly correlated in within-participants designs. In fact, the higher these correlations are, the higher the increase in statistical power compared to between-participants designs (e.g., Lakens, 2013). That is, it is for measures highly correlated across conditions (as is typically the case for mean RTs in different conditions of an experiment), where within-participants designs play out their full strength and differ most from between-participants designs.

### Method

Based on these considerations, we simulated two sets of data, one with a variation in drift rate *v* (“real” effect^5^) and one with a variation in threshold separation *a* (SAT effect) to get a first impression of how LISAS and BIS react to these manipulations. All data were modeled as Wiener diffusion processes (see Ratcliff, 1978; Ratcliff et al., 2016; Ulrich et al., 2015; Vandekerckhove & Tuerlinckx, 2007; Voss & Voss, 2007; Wagenmakers et al., 2007), that is, activation at time *t*, *X*(*t*), is modelled as a scaled Wiener process with a time-independent drift rate *v*

$$X(t)=W(t)\cdot\sigma+v\cdot t$$

with a fixed value of the noise parameter σ = 4 (as in Liesefeld & Janczyk, 2019).^6^ A decision is made when the activation, starting at 0.5 · *a* exceeds either the upper threshold *a* (correct) or the lower threshold at zero (error). The time point where this happens is interpreted as the decision time. Time spent on additional processes of encoding and responding is captured via an additional non-decision time parameter, *t*^ER^, which is added to the decision time to yield the overall RT.

In the first simulation, a “real” effect was induced by varying the drift rate between conditions. In this case, we chose *v*_*1*_ = 0.246 and *v*_*2*_ = 0.254 while keeping the threshold separation constant at *a* = 125. In the second simulation, an SAT was induced by varying the threshold separation between conditions. In this case, we chose *a*_*1*_ = 120 and *a*_*2*_ = 130, while keeping the drift rate constant at *v* = 0.25.^7^

Based on these standard parameters, two sources of variability were added to the respective varied parameter. First, interindividual variability was implemented by adding the same value ${\epsilon}_i^{between}$ to both conditions of a simulated participant *i*. Second, to induce error variance (which, in a within-participants design, is the participant × condition interaction), an additional ${\epsilon}_{i,j}^{within}$ was added to each condition *j* (*j* ∈ {1, 2}) of each participant *i*. Thus, for a participant *i* in condition *j*, the parameter *μ*_*i*, *j*_ (i.e., drift or threshold separation) used for the simulations is the following sum:

$${\mu}_{i,j}={\mu}_j+{\epsilon}_i^{between}+{\epsilon}_{i,j}^{within}$$

The (error) terms ${\epsilon}_i^{between}$ and ${\epsilon}_{i,j}^{within}$ were drawn from a set of random variables ${\boldsymbol{E}}^{between}\sim N\left(0,{\sigma}_B^2\right)$ and ${\boldsymbol{E}}_j^{within}\sim N\left(0,{\sigma}_W^2\right)$, respectively. For the drift rate simulation, we set ${\sigma}_B^2={0.01}^2$ and ${\sigma}_W^2={0.005}^2$; for the SAT simulation we set ${\sigma}_B^2={20}^2$ and ${\sigma}_W^2={10}^2$. Note that the theoretical correlation of the parameters between the two conditions across participants can be calculated as

$$r=\frac{\sigma_B^2}{\sigma_B^2+{\sigma}_W^2}$$

and is accordingly *r* = .80 for the chosen values (see Appendix 2 for a proof). The non-decision time *t*^ER^ was drawn separately for each participant *i*, but was the same for both conditions *j* with ${\boldsymbol{t}}_i^{ER}\sim N\left(\mathrm{300,20}\right)$, thus adding extra between-participants variance in mean RTs. Both simulations were repeated to yield 1000 experiments with *n* = 20 participants each and 1000 trials per condition (i.e., we simulated 2 × 1000 × 20 × 1000 = 40 million individual diffusion processes in total).

### Analyses

In our simulations, raw data were aggregated at the end of each simulated experiment to improve computational efficiency. In this course, the statistics required to calculate BIS, LISAS, and LISAS^cond^ as detailed above were obtained and stored (mean correct RTs and PCs for both measures, and the respective across-trial SDs for LISAS [including all trials of a participant and separately per participant × condition cell; only correct trials were included for RT SDs]). For each of the 1000 experiments, a paired-sample *t* test was calculated between the two conditions on each obtained dependent variable (mean RT, PC, BIS, LISAS) and the percentage of significant results (at α = .05) was recorded. In addition, the effect size ${d}_z=\frac{t}{\surd n}$ was calculated per experiment and averaged across experiments.

### Results

The means, effect sizes, and percentages of significant *t* tests for the drift rate and the SAT simulation are summarized in Table 1. Four aspects of these simulated data are of major relevance here:

**Table 1:** Means of mean RT, PC, BIS, and versions of LISAS, complemented by mean effect size *d*_z_, and the percentage of significant paired *t* tests (at α = .05) when a “real” effect was implemented via different drift rates while keeping the threshold separation constant at *a* = 125 (upper part) or when an effect on SATs was implemented via different threshold separations while keeping the drift rate constant at *v* = 0.25 (lower part)

Measure	Mean 1	Mean 2	Effect size *d*_*z*_	% significant
“Real” effect (*v*_*1*_ = 0.246 vs. *v*_*2*_ = 0.254)				
mean RT	499	496	0.69	79.7
PC	0.88	0.89	–0.78	87.8
BIS	–0.374	0.374	–0.96	96.4
LISAS	556	550	1.1	99.3
LISAS^cond^	556	550	0.9	96.3
LISAS^BIS^	719	705	0.96	96.4
SAT effect (*a*_*1*_ = 120 vs. *a*_*2*_ = 130)				
mean RT	487	510	–0.73	85.0
PC	0.87	0.89	–0.67	79.7
BIS	–0.009	0.009	–0.06	6.7
LISAS	545	561	–0.73	85.0
LISAS^cond^	539	564	–0.73	85.0
LISAS^BIS^	679	678	0.06	6.7

LISAS^BIS^ is introduced and discussed further below, but reported here already for ease of comparison

First, the data of both simulations produced positive correlations between the two conditions; they thus correspond to typical observations in within-participants designs. More precisely, for the drift rate simulation, the mean correlation^8^ for the drift rates (range in square brackets) was *r* = .811 [.309; .960], for mean RT *r* = .979 [.869; .995], and for PC *r* = .684 [−.198; .948]. Similarly, for the SAT simulation, the mean correlation for the threshold separations was *r* = .807 [.282; .974], for mean RT *r* = .831 [.310; .975], and for PC *r* = .793 [−.295; .965].

Second, as becomes evident from Table 1, our manipulations of drift rate and threshold separation across conditions yielded “real” effects and effects on SATs, respectively, with the former indicated by opposing trends and the latter indicated by same-directional trends in mean RT and PC.

Third, when considering BIS and the various versions of LISAS with regard to the “real” effect in Table 1, it appears that all combined measures yielded more significant *t* tests than either mean RT or PC and thus can potentially increase the statistical power when an effect is distributed across mean RT and PC.

Fourth, and most importantly for the present purposes, are the results for BIS and LISAS with regard to the SAT effect in Table 1 (lower part). Remember that variations in mean RT and PC were only due to varying the SAT setting by manipulating the threshold separation parameter *a* in the underlying simulation. While the percentage of significant *t* tests on LISAS and LISAS^cond^ is around the same as for mean RT, this percentage is strikingly reduced for BIS (and LISAS^BIS^, which is designed to mimic BIS and is introduced and discussed further below), namely from 85% (mean RT) or 79.7% (PC) to 6.7% (BIS).

To make sure that the relative insensitivity of BIS to variations in threshold separation is not just a chance finding related to the specific parameters used, we ran additional simulations with other values to cover a broader range of parameters, while focusing only on SAT effects, that is, variations in threshold separation *a* (see Table 2). These simulations yield the same conclusions as those reported in Table 1.

**Table 2:** Additional simulations with SAT effects (for details, see Table 1)

Measure	Mean 1	Mean 2	Effect size *d*_*z*_	% significant
Case 1: *v* = 0.35, *a*_*1*_ = 110 vs. *a*_*2*_ = 130				
mean RT	438	472	–1.46	100
PC	0.92	0.94	–1.18	100
BIS	–0.22	0.22	–0.11	10.4
LISAS	474	496	–1.47	100
LISAS^cond^	467	500	–1.45	100
LISAS^BIS^	547	545	0.11	10.4
Case 2: *v* = 0.35, *a*_*1*_ = 115 vs. *a*_*2*_ = 125				
mean RT	447	464	–0.73	83.7
PC	0.93	0.94	–0.63	73.2
BIS	–0.015	0.015	–0.08	7.9
LISAS	479	490	–0.72	83.7
LISAS^cond^	475	492	–0.72	83.3
LISAS^BIS^	547	546	0.08	7.9
Case 3: *v* = 0.11, *a*_*1*_ = 200 vs. *a*_*2*_ = 220				
mean RT	860	951	–1.45	100
PC	0.80	0.82	–1.29	100
BIS	0.009	–0.009	0.07	11.0
LISAS	1094	1161	–1.44	100
LISAS^cond^	1072	1176	–1.43	100
LISAS^BIS^	1715	1717	–0.07	11.0
Case 4: *v* = 0.11, *a*_*1*_ = 205 vs. *a*_*2*_ = 215				
mean RT	884	930	–0.73	86.7
PC	0.81	0.82	–0.67	79.9
BIS	0.004	–0.004	0.03	6.7
LISAS	1112	1146	–0.73	85.8
LISAS^cond^	1101	1153	–0.72	86.2
LISAS^BIS^	1719	1720	–0.03	6.7
Case 5: *v* = 0.11, *a*_*1*_ = 110 vs. *a*_*2*_ = 130				
mean RT	501	569	–1.41	100
PC	0.69	0.72	–1.34	100
BIS	–0.004	0.004	–0.04	8.2
LISAS	634	690	–1.44	100
LISAS^cond^	608	703	–1.42	100
LISAS^BIS^	1261	1260	0.04	8.2

In sum, both BIS and LISAS maintain “real” effects (and even improve statistical power; Table 1), but—contrary to the conclusions of Vandierendonck (2021b) —only BIS considerably attenuates SAT effects in our simulated within-participants data (Tables 1 and 2). This converges with what Liesefeld and Janczyk (2019) had observed in a much more extensive simulation study for between-participants data. Most importantly for the present purposes, based on these results we can exclude the possibility that the difference between BIS and LISAS observed in our previous study “is quite likely due to the usage of between-subject designs in the Liesefeld-Janczyk paper” (Vandierendonck, 2021b, p. 22). All simulations, analyses, and data used here can be found at: https://osf.io/x9h3n/

## Reanalysis of Vandierendonck (2021b, Exp. 2)

In the previous section, we have arrived at a conclusion diametrically opposed to Vandierendonck (2021b): While we find that BIS is highly effective in attenuating effects that result from mere variations in SATs and that its behavior deviates strongly from that of LISAS, Vandierendonck (2021b) found that BIS and LISAS behave almost identically and neither of them satisfactorily attenuates effects resulting from variations in SATs. To clarify why that is the case, we reanalyzed data from one of his simulations and reviewed the analysis code that is publicly available at 10.5281/zenodo.4593016. This exercise fulfills several additional purposes: It clarifies how BIS is calculated and points out some potential issues with simulating (within-participants) data with the drift-diffusion model, emphasizing the importance of simulating realistic amounts of between- and within-participants variance.

Out of the available data sets, we decided against using the simulation from Vandierendonck’s (2021b) Study 1 (which follows a logic similar to all simulations in Vandierendonck, 2017), because we do not believe that this approach is valid for simulating variations in SAT. Most problematically, in this simulation, the relative size of effects on mean RT and PC is arbitrary (as also discussed in Appendix 1). A non-arbitrary relationship between effects on mean RT and PC is achieved by simulations using the psychologically plausible drift-diffusion model and by manipulating the threshold separation parameter *a*, as was done above and already in Liesefeld and Janczyk (2019). Therefore, we were happy to see that in Study 2 and Study 3, Vandierendonck (2021b) adopted this approach and simulated variations in SAT and difficulty (“real” effects) using the drift-diffusion model. Because the data structure and the underlying reasoning of Study 3 are unnecessarily complex for the present purposes, we decided to work with the data from Study 2.

This study contains 40 (4 PE levels^9^ ×10 speed–accuracy steps) simulated data sets, each with a 2 (drift rate) × 3 (threshold separation) within-participants manipulation. “PE levels” refers to four different sets of drift rate/threshold separation combinations that approximately yielded the desired PEs (.05, .10, .15, and .20) and “speed–accuracy steps” refers to the size of the threshold-separation manipulation in the respective simulated data set. Further details on the simulations can be found in Vandierendonck (2021b). From these data, Vandierendonck extracted (among other measures) mean RT, PE, LISAS, and what we call here BIS^V^ (with “V” standing for “Vandierendonck”) for each of the six cells of each of the 40 studies.

**Fig. 2:**
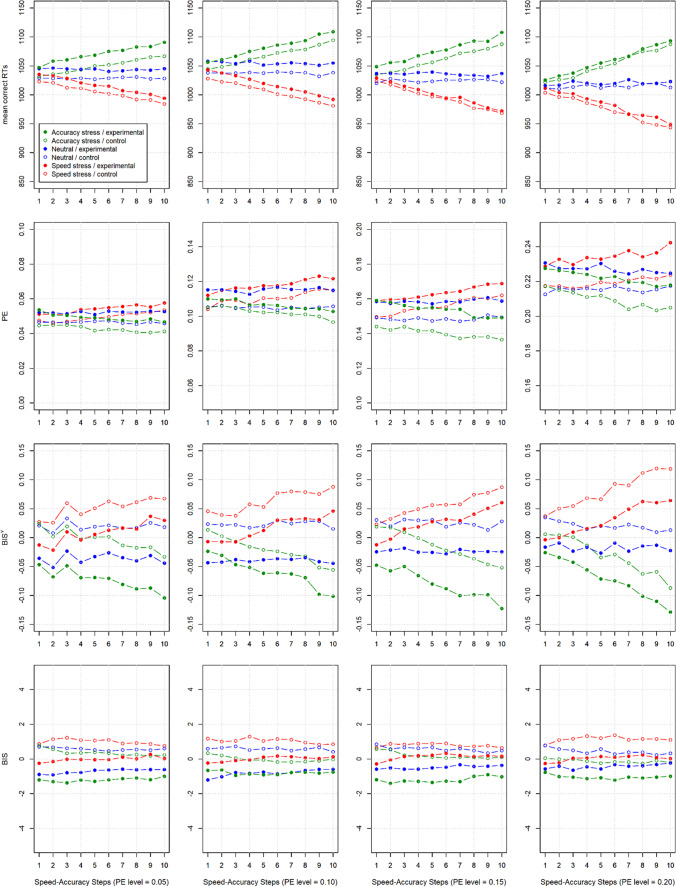
Rows 1–3 reproduce parts of Fig. 4 in Vandierendonck (2021b), recalculated based on the publicly available simulation results and our reading of the analysis code. “BIS^V”^ (row 3) refers to the (erroneous) calculation of BIS in that article. Row 4 presents the pattern for BIS obtained when all required corrections were applied to the calculation. Filled and unfilled circles represent the experimental (lower drift rate) and the control (higher drift rate) condition, respectively. Colors code the three SAT conditions of each simulation and “Speed–Accuracy Steps” refers to the size of the respective SAT manipulation

Surprisingly, at first, we were unable to replicate the pattern for “BIS^”^ as displayed in Vandierendonck’s (2021b) Figures 4–6 with his simulated data (cf. “BIS^V^” and BIS in Fig. 2). Working through his code revealed a programming error (in *getgen.pl*, l. 24–28) that eventually resulted in entering mean *error* RT into the calculation of BIS rather than mean *correct* RT.

An even more consequential, conceptual, problem in the analyses is that instead of using the variance across the participants × condition cells in aggregated mean RT and PC as intended (Liesefeld et al., 2015; Liesefeld & Janczyk, 2019), Vandierendonck (2021b) has used the variance in RTs and accuracies across *trials* to standardize mean RT and PC during the calculation of BIS. Thus, to plot BIS^V^ in Fig. 2, we (incorrectly) used mean error RT and the across-trial variance in error RTs and accuracies, thereby perfectly replicating the “BIS” pattern in Fig. 4 of Vandierendonck (2021b).^10^

**Fig. 3:**
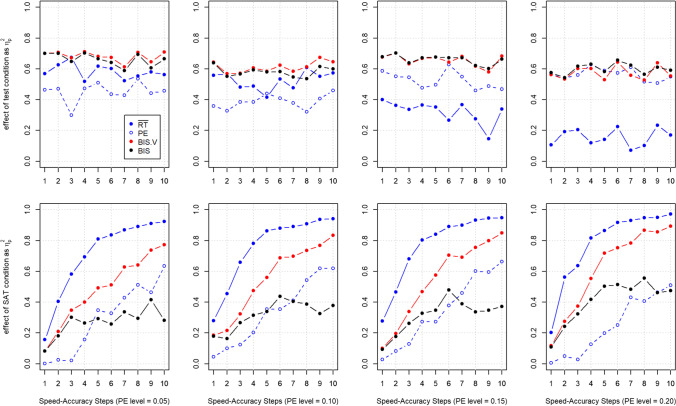
Effect sizes (${\eta}_p^2$) for mean RT, PE, BIS^V^, and BIS for the data of Study 2 of Vandierendonck (2021b). ${\eta}_p^2$ was calculated as $\frac{SS_{effect}}{SS_{effect}+{SS}_{error}}$ and slightly deviates from the ${\eta}_p^2$ reported in Vandierendonck (2021b)

**Fig. 4:**
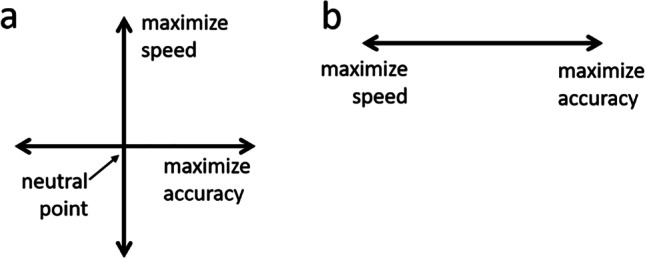
Incompatible conceptions of SATs. Panel **a** illustrates the conception as, in our understanding, underlying Vandierendonck (2017, 2018, 2021b); panel **b** illustrates the conception underlying the present work (as well as that of many others)

When correcting for these mistakes, BIS attenuates SAT effects to a higher degree than all competing evaluated measures and it seems almost unaffected by the size of the threshold separation manipulation in the simulations (i.e., by the “Speed–Accuracy Steps”; see Fig. 2, row “BIS”). However, as discussed further below, the simulated SAT effect still affects BIS (to a higher degree than in our analyses above or in the more extensive between-participants simulations of Liesefeld & Janczyk, 2019), as evidenced by the difference between the colored lines in Fig. 2 and the moderate effect sizes as visualized in Fig. 3.

These observations must be interpreted with some caution, due to various potentially non-ideal choices in Vandierendonck’s (2021b) simulations as detailed in turn. First, in contrast to our simulations above, all variance contributing to the error term of the statistical within-participant tests in Vandierendonck’s (2021b) simulations comes from the stochastic diffusion process itself rather than being explicitly controlled. This variance can be controlled by drawing parameters from a random distribution for each participant × condition cell of the design as done in the above simulations (${\epsilon}_{i,j}^{within}$).

More problematically, the data does not contain sufficient between-participants variance (${\epsilon}_{i,j}^{between}$; reflecting, e.g., pre-experimental variation in ability). While not mentioned in the manuscript, a close inspection of the simulation code reveals that for each participant a random value was drawn from a normal distribution with *M* = 0 and *SD* = 0.001 and this value was added to the drift rate and threshold separation paramete﻿r﻿. That the induced between-participants variance might not be realistic in the data simulated by Vandierendonck (2021b) can be seen by considering that interindividual differences that are stable across experimental conditions result in correlations between conditions, because a participant who responds relatively fast in condition A will also respond relatively fast in condition B. However, in contrast to typical within-participants data (e.g., Lakens, 2013), the correlation between conditions in the data set reanalyzed here is almost zero on average (see Table 3). Thus, unfortunately and in contrast to our simulations reported above, the data simulated by Vandierendonck (2021b) are not representative of within-participants data, despite the purpose of that study to evaluate measures combining speed and accuracy in *within-participants designs*.

**Table 3:** Average correlations (and their range across speed–accuracy steps in square brackets) between the two drift rate conditions for mean RT and PE of Study 2 in Vandierendonck (2021b)

PE level	SAT condition			
	Speed	Neutral	Accuracy	Average
mean RT				
.05	.07 [−.07; .28]	−.07 [−.23; .05]	.17 [.08; .32]	.06
.10	.04 [−.33; .12]	.13 [−.14; .28]	−.11 [−.34; .13]	.00
.15	.00 [−.28; .17]	−.02 [−.29; .20]	.02 [−.23; .20]	.00
.20	.11 [−.14; .27]	.07 [−.18; .27]	.13 [−.12; .28]	.10
PE				
.05	−.03 [−.16; .18]	.06 [−.12; .32]	−.03 [−.17; .06]	.00
.10	.10 [−.04; .26]	−.11 [−.28; .08]	.16 [−.17; .44]	.05
.15	.03 [−.15; .26]	.19 [−.15; .38]	.14 [−.01; .33]	.12
.20	.01 [−.11; .13]	−.08 [−.34; .07]	.23 [.04; .37]	.05

Equally problematic—in particular with regard to BIS—is a potential consequence of drawing only one value per participant and adding it to both the drift rate and the threshold separation parameters: An increase in drift rate decreases RTs and PEs, whereas an increase in threshold separation increases RTs and decreases PEs. Therefore, if drift rate and threshold separation increase in parallel, mean RTs remain relatively stable, while PEs decrease much more; if drift rate and threshold separation decrease, mean RTs remain relatively stable, while PEs increase much more. Thus, by adding the same value to both parameters, more between-participants variance in PEs is induced than in mean RTs. As this variance goes into the denominator of the *z* standardization in the calculation of BIS, any such-induced between-participants variance diminishes the influence of PE on the final BIS score (as if PE was down-weighted). Thus, artificially adding the same term to drift rate and threshold separation parameters to induce between-participants variance exaggerates RTs in the calculation of BIS. The reason why this is not so dramatically the case in the simulated data (see Figs. 2 and 3) is that insufficient between-participants variance was induced in the first place. Note that this is not an issue with BIS, but an issue with the assumption in Vandierendonck’s (2021b) simulations that participants with a high drift rate necessarily also apply a high threshold separation.

Another issue is that Vandierendonck (2021b) simulated only a single experiment per data point in Figs. 2 and 3, so that the resulting data are unlikely to be representative of all possible data sets that could have been generated with the respective employed parameter set. This results in the jagged shape of the curves in Figs. 2 and 3, where, for example, PE can rise or fall with an increase in threshold separation (“Speed–Accuracy Steps”) due to quasi-random fluctuations in the simulations. The individual points in such a graph would become more representative of all potential simulation outcomes by simulating a large number of experiments per parameter combination and then averaging across these simulated experiments as done in our simulations above and in Liesefeld and Janczyk (2019).

Finally, based on these data one could get the impression that just analyzing PE is the best way to handle variations in SAT, because, overall, PE was the measure least affected by variations in threshold separation (in contrast to the effects of variations in threshold separation on PC observed in our simulations, see Tables 1 and 2), while being rather sensitive to variations in drift rate, in particular for high PE levels (when there is room for effects on PE; see Figs. 2 and 3). This unrealistic insensitivity of PE to variations in threshold separation (in part) explains the relatively bad performance of BIS with regard to attenuating variations in SAT (which is still better than the other combined measures and mean RT): if—as is the case in the data simulated by Vandierendonck (2021b)—there is insufficient corresponding variation in PE, variation in mean RT induced by differential SATs cannot be compensated for by any combined measure (see also the section on “Comparisons of three conditions using ANOVAs” and on “Transforming the constituents” in Liesefeld & Janczyk, 2019).

In sum, due to these various issues with the analyses and simulations in Vandierendonck (2021b), for the time being, we recommend referring to our preliminary simulations and analyses above with regard to the question of whether combined speed–accuracy measures can attenuate effects resulting from variations in SAT in within-participants designs, the tentative answer being that BIS can, at least for pairwise comparisons. More comprehensive simulations are desirable, but would overly extend the present article. Furthermore, our reanalyses and comments on the simulation hopefully convey several crucial points in the simulation of within-participants data, and prevent future users of BIS from committing the same mistakes in their calculation of BIS.

## The crucial difference between LISAS and BIS

Having established that BIS and LISAS differ in their behavior and—taking also the extensive simulations and analyses in Liesefeld and Janczyk (2019) into account—that only BIS attenuates spurious effects that are due to differential SATs, we now turn to the question of what differentiates the two measures. While Vandierendonck (2021b) stresses that BIS scores cannot be compared across experiments as a major difference to LISAS^11^, the above reanalyses of his data set indicate that the choice of the variance used for standardization matters most. To see where the opposing views come from and to support users of combined measures to make an informed choice, the following dwells on these two characteristics in some detail. Following these theoretical considerations, we will demonstrate that indeed variance in standardization rather than the different scaling matters most. In particular, by using BIS’ standardization variance, we can easily modify LISAS, so that it attenuates the effects of differential SATs while maintaining “real” effects in our simulated data, just like BIS does.

On the surface, BIS is indeed highly similar to LISAS (as demonstrated in Appendix A of Vandierendonck, 2021b). This superficial similarity is not surprising, because both measures combine mean RT and PC/PE by first bringing them to the same scale. Which scale they are brought to is, we would argue, a relatively arbitrary choice that is non-consequential for the measure’s behavior (as already discussed in Liesefeld & Janczyk, 2019, p. 50). LISAS is scaled in terms of RTs and, according to Vandierendonck (2021b), “can be interpreted as an RT corrected for errors” (p. 24). Liesefeld and Janczyk (2019) suggested (but by no means prescribed) scaling BIS in terms of above-average (BIS > 0) or below-average (BIS < 0) performance across participants and conditions in the analyzed experiment, with higher absolute values reflecting stronger deviation from the average. To us, this appeared to be the most interesting scaling, because absolute RTs are typically not in the focus of psychological studies and comparisons of absolute performance across studies is not usually desired or even possible, because absolute performance is affected by many incidental choices regarding stimuli and experimental designs that would differ between studies. Rather, experimental research usually focuses on performance differences between conditions (and maybe participants) within an experiment, which is directly reflected in BIS with the scaling suggested by Liesefeld and Janczyk (2019).

Having said this, if, for whatever reason, a scaling in terms of RTs (like for LISAS) is desired, BIS can easily be rescaled accordingly (Liesefeld & Janczyk, 2019, p. 50):3where ${S}^{\overline{RT}}$ refers to the SD of mean RTs used in the calculation of BIS (usually, across all participant × condition cells) and  refers to the grand mean RT, that is, mean RTs averaged across all conditions and participants. Such linear transformations do not affect the behavior of BIS in any way (see Appendix 3 and Liesefeld & Janczyk, 2019, Footnote 9). Actually, on its first application (before it even got its name), BIS was scaled and interpreted as mean RT corrected for errors (Liesefeld et al., 2015; as pointed out in Liesefeld & Janczyk, 2019, Footnote 3).

By contrast, which variance is used for standardization is crucial: BIS uses the variance across the data points of interest. In typical experimental designs of the type simulated here, these data points are mean RT and PC, that is, the aggregated data. The underlying idea is to combine mean RT and PC within one score (BIS) so that both constituent measures (mean RT and PC) contribute the same amount of variance to this score (i.e., correlate with it to the same degree; see Liesefeld & Janczyk, 2019, pp. 45–46). For this goal, it is of no direct relevance how raw RTs (and accuracies) are distributed across trials, but the distribution of the derived measures (mean RT and PC per participant × condition cell) that are actually submitted to standard statistical tests (e.g., ANOVAs or a *t* tests) is what counts. That the distribution of means differs from the distribution of the raw data is probably most widely known for RTs: While distributions of raw RTs are heavily left-skewed (have a long right tail), the distribution of mean RTs more closely approximates a (symmetric) normal distribution if a sufficient number of trials is aggregated. Typically, the best estimate of the variance of the aggregated measures is achieved by calculating it across all participant × condition cells, but there are situations where it is desirable to equate BIS across two or more groups of participants (e.g., when the focus is on a group-by-condition interaction; see Liesefeld et al., 2015). We cannot readily see, nor did we find any respective discussion in Vandierendonck’s publications, as to why it is desirable to scale aggregate measures by across-trial variance as done for LISAS.

To demonstrate that the choice of the standardization variance is crucial, we tweaked LISAS so that it mimics the behavior of BIS as a result. This is done by simply replacing the across-trial variance of raw performance used to scale PE by the across-cell variance of the aggregated data (as used by BIS):

4 $$LISAS_{i,j}^{BIS}=\overline{RT_{i,j}}+\frac{S^{\overline{RT}}}{S^{PE}}\cdot PE_{i,j}$$

Please compare Eq. 4 to the original version of LISAS (in our notation) in Eq. 2 and note that we merely adapted the term for scaling *PE*_i,j_. As shown in Tables 1 and 2, LISAS^BIS^ indeed strongly attenuates effects from differential SATs while maintaining “real” effects, just like BIS does. Finally, Appendix 3 demonstrates that LISAS^BIS^ is essentially a version of BIS linearly transformed to the scale of mean RTs (LISAS^BIS^ = BIS^RTscaled^ + *C*), taking—in contrast to Appendix A of Vandierendonck (2021b), which is based on a single participant—also the crucial standardization variances into account.

## Are combined performance measures needed at all?

To us, the major aim of combined performance measures is to integrate measures of speed (mean RT) and accuracy (PC) in a way that attenuates SAT effects while maintaining “real” effects. The same goal can be achieved by fitting cognitive models such as the drift-diffusion model (i.e., the very model used here for simulating data) to empirical data and then analyzing the parameter estimates that are considered to reflect “real” effects. In fact, the drift rate of the drift-diffusion model closely corresponds to what BIS is assumed to reflect and, in a way, calculating BIS here and in Liesefeld and Janczyk (2019) can be conceived of as recovering effects on the drift rate parameter from the simulated data. Thus, fitting the drift-diffusion model to each individual cell of the design and submitting the drift rate estimates to further statistical tests (as has been done before; e.g., Janczyk & Lerche, 2019; Schuch, 2016) would achieve the same purpose as calculating BIS. In fact, the modeling approach is far superior in many ways (e.g., Ratcliff et al., 2016). For example, it provides estimates of many additional parameters and allows to impose useful constraints on parameter estimates (e.g., Vandekerckhove & Tuerlinckx, 2007) and to directly test psychological theories by comparing different models (e.g., Koob et al., 2021). Furthermore, an estimate of some basic parameters of the drift-diffusion model has been suggested that is equally easy to apply as BIS (Wagenmakers et al., 2007; which is not without critiques, though, Ratcliff, 2008). Clearly, the purpose of BIS is not to replace this powerful approach, but to offer an alternative in cases where model fitting does not seem applicable. The two approaches complement each other, because decision models such as the drift-diffusion model assume a very specific set of cognitive processes and, in particular, that SAT effects reflect variation in the decision criterion. Whenever the model assumptions are likely to apply to the psychological phenomenon under investigation, this specificity is desirable. By contrast, BIS is constructed based on purely statistical considerations, namely equal weighting of the two constituent measures, mean RT and PC, and does not make any assumptions with regard to the underlying cognitive processes. We expect BIS to be useful as long as psychological phenomena are investigated for which there is no easily accessible model that can be used instead or whenever there is doubt in the validity or applicability of these models (see also Liesefeld & Janczyk, 2019, pp. 52–53).

Another consideration that would, in our opinion, render combined measures largely dispensable was brought forward by Vandierendonck (2021b), who argues that differential SATs were impossible when trials from the various experimental conditions are randomly intermixed in within-participants designs and therefore recommends to use such designs, rather than combined performance measures, in order to avoid the issues with potential condition-specific variation in SAT. If this was true, it would indeed resolve the issue of differential SATs and, thus, neither combined measures nor model fitting would be needed for that purpose. Problematically, however, (a) such random intermixing is not always possible or desirable and (b) it is an empirical question whether intermixing makes differential SATs impossible that, we believe, must be tested for each specific situation.

Regarding point (a), many research questions require across-group comparisons, such as those involving different age groups or the comparison of intervention and control groups. Furthermore, even in within-participants designs, random intermixing is not always possible or desirable. An example close to our own work is response-effect compatibility in the action-control literature (Janczyk & Lerche, 2019; Kunde, 2001), but there are many further reasons that might prevent an experimenter from intermixing experimental conditions of interest in a fully random fashion. 

Regarding point (b), as powerful as this technique might be, random intermixing does not guarantee the absence of differential SATs. For example, it seems likely that in tasks with longer mean RT, participants decide that they have spent sufficient time on a given, particularly difficult trial and respond prematurely in a higher number of cases than on easy trials (e.g., Liesefeld et al., 2015, where difficult mental rotations were randomly intermixed with easy mental rotations). Such behavior could, for example, be based on a time-out strategy. Also, an adaptation of SATs based on a preliminary scanning of the stimulus does not seem too unrealistic after all. Consider for example a visual search task with a strong difference in difficulty between randomly intermixed inefficient search and efficient search conditions. All else being equal, participants might be less willing to spend much time on the inefficient search trials but rather tend to make their decision based on less evidence and proceed with the next (probably easier) trial prematurely. A coarse and preliminary scan of the scene can often tell whether a search display is difficult or easy (e.g., whether non-targets are homogeneous or heterogeneous, see Liesefeld & Müller, 2020) and result in a trial-wise adaptation of the search strategy (e.g., Tay et al., 2022). Another example are intertrial effects, that is, the observation that features of a preceding trial affect performance on the current trial, potentially by changing SATs. As a matter of fact, intertrial effects on the threshold parameter have been observed previously (e.g., Schuch, 2016). In sum, in contrast to the viewpoint expressed in Vandierendonck (2021b), we argue that the intermixing technique does not generally solve the issue of condition-specific SATs and we believe that combined performance measures remain useful for this purpose.

## Appendix 1: Incompatible conceptions of speed–accuracy trade-offs

In order to examine and discuss SATs it is certainly useful to agree on a common definition of what an SAT actually is. Unfortunately, Dr. Vandierendonck seems to use a definition that is incompatible with the one that we (and many others, as we will demonstrate below) hold. In fact, it is difficult for us to fully grasp the definition of SATs in Vandierendonck (2017, 2018, 2021b) and therefore the best thing we can do in order to achieve progress in the debate is to explain in considerable depth how we conceive of SATs and variations therein and why the alternative conception does not make sense to us. These differences in definitions obviously have implications for how SATs should be simulated and for the criteria that determine whether a combined measure handles SATs well (or whether these measures should handle SATs at all; see Vandierendonck, 2021b, pp. 23–24). We assume that this appendix is of interest for only very few readers: those who were confused by the way we simulated or discussed SATs in the main article and those who were confused by the respective aspects of Vandierendonck (2017, 2018, 2021b) and want to find out where that uneasiness comes from.

From various interactions, including careful reads of his works and reflections on his simulations, we believe that Dr. Vandierendonck thinks of SATs as two independent dimensions, (1) increase or decrease speed and (2) increase or decrease accuracy, with a true neutral point where none is either increased or decreased. This would be best illustrated by a Cartesian coordinate system (Fig. 4a), where the “neutral point” is the origin. By contrast, we think of SATs as a single continuum with the poles “maximize accuracy” and “maximize speed” (Fig. 4b). Notably, no “neutral point” exists in this case: Participants must in any case trade one aspect of performance for the other. Even if they chose a point just in the middle between the two poles, this would still be a trade-off. A useful analogy might be a car that has only a limited amount of fuel. The driver must at any moment decide whether to drive fast and therefore cover only a short distance or to drive slow and therefore reach a more distant goal (with a given amount of fuel). There is no neutral point of driving fast without sacrificing range or driving far without sacrificing speed.^12^

Given this fundamental difference in viewpoints, disagreements on many points regarding the simulation of SATs and the evaluation criteria that should be applied to combined performance measures are inevitable. However, clearly elaborating the crucial disagreement allows the reader to decide for one or the other viewpoint and therefore to decide whether to put trust in our results and interpretations or in those of Vandierendonck (2017, 2018, 2021b)

Contemplating on where the conception of SATs displayed in Fig. 4a could come from, we presume the following train of thought and put it in italics to clearly demarcate it from our standpoint: *If instructions do not emphasize either speed or accuracy, participants adopt the neutral point with no SAT, that is, neither is speed traded for accuracy nor is accuracy traded for speed. If now in addition to this baseline condition with neutral instructions, another condition stresses the importance of speed, participants will respond faster; if a third condition stresses accuracy, participants will perform more accurately.*

Of course, one can come up with other manipulations instead of instructions (e.g., payoff schemes, time pressure) that would have similar effects on SATs, but it is useful to bear with this example just to have something specific to talk about. As it stands, these thoughts on SATs seem reasonable and are in line with Fig. 4a. So, why are we not convinced by this conception of SATs?

First, it is easy to see that “participants respond faster” misses the empirical fact that with these instructions, participants will also respond less accurately and, respectively, “participants respond more accurately” misses the empirical fact that participants will then also respond more slowly (see the driving-fast-or-far analogy above). Therefore, when Vandierendonck (2017) simulates variations in SAT by independently manipulating mean RT or PE, he creates data that, in our opinion, do not comply with reality. Even when possible, increasing speed without sacrificing accuracy or vice versa (as in the mentioned simulations) does not reflect an SAT proper as we conceive of it, but would require some extra processing capacity (e.g., extra effort; Kahneman, 1973). The issue becomes even more evident when adding a fourth condition (that is also part of Vandierendonck’s, 2017, simulations): *…if a fourth condition stresses both speed and accuracy, participants will perform faster and more accurately.*

Second, instructing participants to equally weight speed and accuracy and what participants actually do are two different things. It appears unlikely to us that participants can somehow balance responding fast and responding accurately like two children can justly share a piece of cake by dividing it exactly in half. Quite the opposite: participants have no way of objectively judging how much gain in speed is worth how much loss in accuracy, because the two are fundamentally different aspects of performance that cannot readily be compared by the same yardstick. Again, this becomes clear when using the driving analogy: A driver cannot justly share the fuel to obtain comparable values of speed and distance, because speed is measured in miles/h and distance is measured in miles, and there is no objective transformation between the two (let alone that participants would know this transformation and be able to apply it on the fly). Sure, the driver could drive fast until half of the fuel is used and then maximize distance with the second half of the fuel, but such sequential strategies are not possible for performance on a single trial of an experiment and therefore overstrain the analogy. Attesting to the hypothesis that participants cannot simply adopt any desired SAT (such as a neutral point), it has been shown that experimental manipulations (such as instructions) designed to manipulate SATs can affect parameters of the drift-diffusion model beyond the threshold parameter (e.g., Katsimpokis et al., 2020); the reasons for this mismatch might be found in how participants react to the experimental manipulations as well as in the assumptions of the drift-diffusion model with current evidence favoring the former possibility (Lerche & Voss, 2018).

Third, one may still declare that the behavior participants produce under “neutral” instructions (assuming that instructions can be neutral) is the “neutral point.” Importantly, this “neutral point” must still lie somewhere on the continuum in Fig. 4b (likely at different points for different participants). Even if the point was just in the middle of the two extremes (which is quite unlikely for the reasons discussed above), it is still not really neutral, because participants still commit to a certain relative weighting of speed and accuracy; in other words, they decide on a trade-off between speed and accuracy in each single condition (that appears fair or in line with task instructions to them as far as they feel able to judge this at all).

Assuming the absence of a “neutral point,” one might wonder how experimental psychologists can even attempt to “control for SATs” or to “rule out that an observed pattern of results is due to an SAT.” The answer is that these statements indeed do not make any sense if taken literally and should be interpreted as abridgments for “control for variations in SAT” and “rule out that the observed pattern of results is only due to differential SATs.” In fact, we recommend using the latter, more accurate phrasings in future papers. The goal is not to avoid SATs, but to make sure that the same SAT is used in all conditions or to transform the data in a way that the SAT is statistically constant across all conditions (in the sense of partializing out a variable such as age to render that variable statistically constant rather than removing it, i.e., producing “age-free” participants). The latter is what, in our opinion, combined performance measures are supposed to do.

While the simulations of Vandierendonck (2017) rely on the independent-dimensions conception of SATs in Fig. 4a, the diffusion model, by varying the threshold separation parameter *a*, implements the continuum view illustrated in Fig. 4b. Indeed, varying the threshold separation parameter is the standard way of simulating SATs in the diffusion model employed by many experts in the field (e.g., Dutilh et al., 2012; Hedge et al., 2018a, b, 2021; Lerche & Voss, 2018). Therefore, we appreciate seeing that Vandierendonck (2021b) now uses the drift-diffusion model in his Study 2 and Study 3, thus at least partially adopting our conception of SATs. Such a partial adoption of our conception is also evident in his Study 1, where he varies mean RTs and PEs concurrently with opposing sign. The issue with his Study 1 is that the relative size of the mean RT and PE variation is fully arbitrary and therefore not representative of real data or useful for examining how combined measures handle variations in SATs (an issue that applies to all simulations in Vandierendonck, 2017).

Thus, the simulations in Vandierendonck (2021b) seem to comply more with the conception of SATs depicted in Fig. 4b than that depicted in Fig. 4a. There are at least two indications that the view depicted in Fig. 4b is not fully adopted though: (a) Vandierendonck (2021b) still clings to the notion of some “neutral point”; and (b) Vandierendonck (2021b) claims that in between-participants designs, “the increased speed by one subject may be compensated by the increased accuracy of another subject” (p. 6) and that this “issue” would somehow invalidate BIS (p. 4). Perhaps this last claim assumes that the first participant can increase speed without sacrificing accuracy and the second participant can increase accuracy without sacrificing speed by merely adjusting the SAT. Without these assumptions, we do not see any issue here.

On this background, we can now try to work out which conception of SATs other researchers hold. Although the dimensionality of SATs or the existence of some “neutral point” is hardly ever a topic in the literature, we compiled a list of statements from papers on various research questions that will conclude this appendix. These authors do not—at least according to our reading—conceive of SATs as consisting of two independent dimensions or as having a neutral point as in Fig. 4a, but would likely subscribe to the continuum view in Fig. 4b, and some of these statements even explicitly mention a “speed–accuracy continuum” (emphases added):

- “People can often control their level of SAT, that is, select or change *their position along a continuum of speed versus accuracy*” (Rinkenauer et al., 2004, p. 1)
- “to examine the mechanisms by which people control *their position along an SAT continuum*” (Osman et al., 2000; Abstract)
- "...because *speed and accuracy are inversely related* [...]. It is not unlikely, therefore, that subjects try to find *some reasonable compromise or tradeoff between these competing objectives*" (Adam, 1992, p. 174)
- “Under time pressure, it is usually *not possible to respond quickly and accurately at the same time*. Therefore, people *must trade speed for accuracy*...” (Hübner et al., 2021, Abstract)
- “Decision threshold is thought to map onto a person’s *decision strategy regarding their speed–accuracy tradeoff*, where participants can either *raise their threshold to respond more slowly with greater accuracy, or lower their threshold to respond more quickly with lesser accuracy*.” (Evans, 2021, p. 2)
- "The accuracy group produced very accurate but slow movements, whereas the speed group produced very fast but inaccurate movements. This speed–accuracy tradeoff phenomenon was statistically confirmed by a *strong negative between-subject correlation* between movement time and variable error (r = −.84)" (Adam, 1992, p. 175)
- "From Figure 5, it is clear that *movement time and VE are indeed inversely related*, such that subjects trade movement speed for endpoint accuracy *to form a speed–accuracy tradeoff continuum*." (Adam, 1992, p. 180)
- “There remains, however, a concern that SART [sustained attention to response task] performance might, in part, reflect strategic choices in responding along a speed–accuracy trade-off curve [...]. One of the more venerable observations of experimental psychology is that *errors tend to increase with response speed* (Woodworth, 1899).” (Seli et al., 2012).
- “What accounts for the trade-off relation between the two main components of fluency (speed and accuracy) so that we can *generate behavior more rapidly only at the expense of a higher probability of error*...” (MacKay, 1982, p. 483)
- In his comprehensive review of (the history of) SATs, Heitz (2014) writes, “Outside of this asymptotic performance lay *a nether region of neither wholly accurate nor wholly fast*” (pp. 1–2), certainly also implying a continuum (with the poles “wholly accurate” and “wholly fast” but without any neutral point).

## Appendix 2: Correlation of parameter values across participants

In the simulation, we defined the respective parameter of a particular participant *i* in condition *j* as

$${\mu}_{i,j}={\mu}_j+{\epsilon}_i^{between}+{\epsilon}_{i,j}^{within},$$

where ${\epsilon}_i^{between}$ and ${\epsilon}_{i,j}^{within}$ (*j* ∈ {1, 2}) are realizations of a set of independent random variables [${\boldsymbol{E}}^{between},{\boldsymbol{E}}_1^{within},{\boldsymbol{E}}_2^{within}$] and *μ*_*j*_ is a constant. Regarding the distribution of these random variables, henceforth referred to as the error terms, we assumed ${\boldsymbol{E}}^{between}\sim N\left(0,{\sigma}_B^2\right)$ and ${\boldsymbol{E}}_j^{within}\sim N\left(0,{\sigma}_W^2\right)$. At the level of random variables, we can therefore define *J* random variables **X**_**j**_ (*j* ∈ {1, 2})

$${\mathbf X}_{\mathbf j}=\mu_j+\boldsymbol E^{between}+\boldsymbol E_j^{within},$$

where each **X**_**j**_ reflects the distribution of simulation parameters in condition *j*.

We now want to derive the expected correlation between the two conditions, that is, the correlation of the random variables

$${\boldsymbol{X}}_{\mathbf{1}}={\mu}_1+{\boldsymbol{E}}^{between}+{\boldsymbol{E}}_1^{within}\mathrm{and}\ {\boldsymbol{X}}_{\mathbf{2}}={\mu}_2+{\boldsymbol{E}}^{between}+{\boldsymbol{E}}_2^{within}.$$

We begin by calculating the covariance as

$$COV\left({\boldsymbol{X}}_{\mathbf{1}},{\boldsymbol{X}}_{\mathbf{2}}\right)= COV\left({\mu}_1+{\boldsymbol{E}}^{between}+{\boldsymbol{E}}_1^{within},{\mu}_2+{\boldsymbol{E}}^{between}+{\boldsymbol{E}}_2^{within}\right)= COV\left({\mu}_1,{\mu}_2\right)+ COV\left({\mu}_1,{\boldsymbol{E}}^{between}\right)+ COV\left({\mu}_1,{\boldsymbol{E}}_2^{within}\right)+ COV\left({\boldsymbol{E}}^{between},{\mu}_2\right)+ COV\left({\boldsymbol{E}}^{between},{\boldsymbol{E}}^{between}\right)+ COV\left({\boldsymbol{E}}^{between},{\boldsymbol{E}}_2^{within}\right)+ COV\left({\boldsymbol{E}}_1^{within},{\mu}_2\right)+ COV\left({\boldsymbol{E}}_1^{within},{\boldsymbol{E}}^{between}\right)+ COV\left({\boldsymbol{E}}_1^{within},{\boldsymbol{E}}_2^{within}\right)$$

Because *μ*_1_ and *μ*_2_ are constants and all three error terms are assumed as being independent (resulting in zero covariances), the covariance reduces to

B1 $$COV\left({\boldsymbol{X}}_{\mathbf{1}},{\boldsymbol{X}}_{\mathbf{2}}\right)= COV\left({\boldsymbol{E}}^{between},{\boldsymbol{E}}^{between}\right)={\sigma}_B^2$$

We continue with calculating the variances of ***X***_**1**_ and ***X***_**2**_ as

B2 $$V\left({\boldsymbol{X}}_{\mathbf{1}}\right)=V\left({\mu}_1+{\boldsymbol{E}}^{between}+{\boldsymbol{E}}_1^{within}\right)=V\left({\boldsymbol{E}}^{between}\right)+V\left({\boldsymbol{E}}_1^{within}\right)={\sigma}_B^2+{\sigma}_W^2$$

and

B3 $$V\left({\boldsymbol{X}}_{\mathbf{2}}\right)=V\left({\mu}_2+{\boldsymbol{E}}^{between}+{\boldsymbol{E}}_2^{within}\right)=V\left({\boldsymbol{E}}^{between}\right)+V\left({\boldsymbol{E}}_2^{within}\right)={\sigma}_B^2+{\sigma}_W^2$$

Using (B1), (B2), and (B3), we can now calculate the correlation between ***X***_**1**_ and ***X***_**2**_ as

$$r_{{\boldsymbol X}_{\mathbf1},{\boldsymbol X}_{\mathbf2}}=\frac{COV\left({\boldsymbol X}_{\mathbf1},{\boldsymbol X}_{\mathbf2}\right)}{\sqrt{V\left({\boldsymbol X}_{\mathbf1}\right)}\cdot\sqrt{V\left({\boldsymbol X}_{\mathbf2}\right)}}=\frac{\sigma_B^2}{\sqrt{\sigma_B^2+\sigma_W^2}\cdot\sqrt{\sigma_B^2+\sigma_W^2}}=\frac{\sigma_B^2}{\sigma_B^2+\sigma_W^2}$$

## Appendix 3: BIS and LISAS^BIS^

As mentioned in the main document, our tweaked version of LISAS, LISAS^BIS^, is essentially a version of BIS linearly transformed to the scale of mean RTs. To see this, we need the formula for BIS scaled to mean RTs (Eq. 3; note that we here use PE instead of PC and want to make higher values stand for worse performance—as is the case for RTs—so that we need to add up the two constituents rather than subtract one from the other; see Liesefeld & Janczyk, 2019, p. 56 and Footnote 3):

expanding and rearranging yields:

$${BIS}_{i,j}^{RTscaled}={\overline{RT}}_{i,j}+\frac{S^{\overline{RT}}}{S^{PE}}\cdot{PE}_{i,j}-\frac{S^{\overline{RT}}}{S^{PE}}\cdot\overline{PE}$$

Note that the last term has no index and therefore is constant and the rest is the formula for LISAS^BIS^ (Eq. 4), therefore:

$$BIS_{i,j}^{RT\;scaled}=LISAS_{i,j}^{BIS}-C$$

As linear transformations do not affect the behavior of the combined measures (see section *The crucial difference between LISAS and BIS* in the main article), the behavior of BIS and LISAS^BIS^ is identical (see Tables 1 and 2).

## Footnotes

1 We suggest using the sample SD for standardization, that is, the SD with *k* in the denominator rather than the square root of the estimate of the population variance with *k* − 1 in the denominator (with *k *referring to the number of data points entered into the calculation; here: *k* = *n* · *j*). Except for very small sample sizes, the effect of using one or the other SD should be negligible though. It might be helpful to reproduce the examplary calculation in Table 1 of Liesefeld and Janczyk (2019). Functions to easily calculate BIS are provided at: https://github.com/Liesefeld/BIS.

2 For clarity, we deviate from previous notations of the formula for LISAS, which have used “*S*_PE_” instead of “*S*^E^” (see Liesefeld & Janczyk, 2019; Vandierendonck, 2017, 2018, 2021b). Strictly speaking, there is no PE for individual trials and thus one cannot calculate *S*^PE^ across trials. “*S*^PE^” really makes sense only for averaged data, such as in the calculation of BIS. Accordingly, we now use “${S}^{\overline{RT}}$” (i.e., with a bar above “RT”), rather than “*S*_RT_” in the formula for BIS to highlight this crucial difference to LISAS.

3 This is the version calculated by Liesefeld and Janczyk (2019), because in their between-participants design, each participant contributed data to only one condition (see Vandierendonck, 2018; but see Vandierendonck, 2021b, p. 22).

4 Defined in this way, the parameter *a* denotes the separation of the lower (erroneous) and the upper (correct) threshold and the starting point is set at *z* = 0.5 · *a*.

5 We are aware that SAT effects are also “real,” but for lack of a better word, we will reserve the term here to refer to effects that are due to between-condition differences in ability or difficulty.

6 To efficiently simulate the decision component, we exploited the R package DMCfun (Mackenzie & Dudschig, 2021), which can efficiently simulate data and fit the Diffusion Model for Conflict tasks (DMC; Ulrich et al., 2015) by using C++ code. For the present purposes, we set the amplitude of the automatic process (modelled as a Gamma function in DMC) to *A* = 0. Note that the noise parameter acts as a scaling parameter affecting the absolute value of the other parameters. In line with the more typical usage, the values of the parameter *a* given here refer to the threshold separation and not to the distance between starting point and threshold as implemented in DMCfun.

7 This choice of parameters is somewhat arbitrary and more extensive simulations are planned for future studies, but the selected parameters fulfill three criteria of relevance for the present study: (1) The mean of two parameters in one simulation is the fixed value in the other simulation to improve the comparability of the two simulations. (2) Mean RT and PC arguably had reasonable values. (3) The percentage of significant *t* tests was below ceiling for all performance measures.

8 Mean correlations were calculated by averaging, across all simulated experiments, Fisher *z-*transformed raw correlations between the two conditions of each experiment and back-transforming the resulting mean value.

9 Here and in the following, it is important to note that PC = 1 − PE. While we decided to use PC (above and in Liesefeld & Janczyk, 2019), Vandierendonck (2017, 2018, 2021b) uses PE. To maintain comparability to Vandierendonck (2021b), we plot and discuss his results in terms of PE.

10 While the present manuscript was under revision, a correction notice to Vandierendonck (2021b) has been published aiming to correct for these mistakes by using variance of the aggregated measures for standardization (Vandierendonck, 2021a). Unfortunately, instead of using correct RTs and the variance across the participants × condition cells in the calculation of BIS as recommended by Liesefeld and Janczyk (2019), p. 42, p. 52; see also their Table 1), incorrect RTs were included and only the between-participants variance was used for standardization. Still, the original error remains instructive for the present purposes, because it illustrates the importance of standardizing based on the variance of the aggregated measures. Issues with the version of BIS calculated in the correction notice seem related to the way this specific set of data was simulated (as discussed below) and are therefore of less general relevance.

11 Vandierendonck (2021b) also claims that with BIS “RT changes by one subject can be compensated by PE changes in another subject” (p. 4), but does not explain this further and we can only speculate that this is related to a different conception of what exactly SATs are (see our Appendix 1).
