# Correction to: Same same but different: Subtle but consequential differences between two measures to linearly integrate speed and accuracy (LISAS vs. BIS)

**DOI:** 10.3758/s13428-022-01963-9

**Published:** 2022-09-01

**Authors:** Heinrich R. Liesefeld, Markus Janczyk

**Affiliations:** Department of Psychology, University of Bremen, Hochschulring 18, D-28359 Bremen, Germany

**Correction to: Behav Res**

10.3758/s13428-022-01843-2

A subtle but crucial mistake was introduced in Eq. 2 during copy-editing: LISAS is calculated using the standard deviation of RTs across trials and conditions (${S}_i^{RT}$). Unfortunately, a bar was added above RT (${S}_i^{\overline{RT}}$), which would refer to the standard deviation of *mean* RTs across conditions. This mistake can actually serve as an instructive reminder, because whether standardization is performed on raw data (LISAS) versus on averaged data (BIS) affects the suitability of the combined measure to control for speed–accuracy trade-offs as demonstrated in the paper.

